# Elevated Serum Small Dense Low-Density Lipoprotein Cholesterol May Increase the Risk and Severity of Coronary Heart Disease and Predict Cardiovascular Events in Patients with Type 2 Diabetes Mellitus

**DOI:** 10.1155/2021/5597028

**Published:** 2021-05-10

**Authors:** Juan Huang, Jun-Xu Gu, Hui-Zhang Bao, Shan-Shan Li, Xiao-Qin Yao, Ming Yang, Yang Li, Ai-Min Zhang, Yue Yin, Na Zhang, Mei Jia, Ming Su

**Affiliations:** ^1^Department of Traditional Chinese Medicine, Peking University International Hospital, China; ^2^Department of Clinical Laboratory, Peking University People's Hospital, China; ^3^Department of Clinical Laboratory, Peking University International Hospital, China

## Abstract

**Background:**

Coronary heart disease (CHD) is a common and severe complication in type 2 diabetes mellitus (T2DM) patients. Increased amount of circulatory small dense low-density lipoprotein cholesterol (sdLDL-C) particles is known to be a sign of dyslipidemia and can result in atherosclerosis. However, the association between serum sdLDL-C levels and CHD in T2DM patients remains unclear.

**Methods:**

A total of 3684 T2DM patients who received selective coronary angiography (CAG) were selected. For analyzing the association between sdLDL-C and CHD severity in T2DM, the patients with CHD were further divided into four subgroups according to the quartiles of sdLDL-C. A multivariate logistic regression was used for analyzing the risks and severity of CHD. A total of 3427 patients with continuous stable CHD were recruited and followed up for 5 years.

**Results:**

Serum sdLDL-C levels in the CHD group were significantly increased compared with those in the non-CHD group [0.80 (0.49) mmol/L vs. 0.70 (0.30) mmol/L, *p* < 0.001]. The results from CHD subgroup analysis indicated that the sdLDL-C levels in patients with multiple-vessel disease and high Gensini score (GS) were significantly increased. By adjusting the confounding factors and analyzing with multiple logistic regression, we found that sdLDL-C independently correlated with the presence and severity of CHD (CHD: OR = 2.257; multiple-vessel disease: OR = 3.288; high GS: OR = 2.554). A total of 484 major cardiovascular events (MACEs) were documented. After Kaplan-Meier analysis and chi-squared analysis, the incidence of MACEs in the high sdLDL-C group was higher than that in the low sdLDL-C group (16.04% vs. 12.25%, *p* = 0.002).

**Conclusion:**

In T2DM patients, elevated serum sdLDL-C may increase the severity of CHD and predict cardiovascular events in the future. Therefore, serum sdLDL-C may be a potential biomarker for the surveillance of CHD in T2DM patients.

## 1. Introduction

Coronary heart disease (CHD) is one of the most common conditions in the world, with high morbidity and mortality [1–3]. Over the past decades, a large number of studies have reported its possible risk factors, such as diabetes, hypertension, dyslipidemia, and smoking, in order to early assess the risk of cardiovascular disease [4, 5]. Type 2 diabetes mellitus (T2DM) occurs mainly in the adults who are obese and is a common metabolic disease characterized by hyperglycemia and insulin resistance [6]. T2DM is associated with dyslipidemia alone or with metabolic syndrome, thereby increasing the risk of cardiovascular disease [7, 8]. Diabetic dyslipidemia is typically characterized by increased levels of low-density lipoprotein cholesterol (LDL-C) and triglycerides (TG) and decreased levels of high-density lipoprotein cholesterol (HDL-C) [9].

Plasma LDL-C is composed of a series of particles with different diameter, density, and chemical compositions [10]. LDL-C with smaller particles and higher density is named as small dense low-density lipoprotein cholesterol (sdLDL-C). On the contrary, LDL-C with larger particles and smaller density is defined as large and light LDL-C, and the subcomponent between them is medium-density LDL-C. Compared with LDL-C, sdLDL-C is considered as more atherogenic [11, 12]. Basic and clinical studies have shown that sdLDL-C is a risk factor for the development of atherosclerosis, and elevated sdLDL-C levels may promote the development of CHD [13, 14]. However, the pathogenic role of sdLDL-C in patients with stable CHD, especially in patients with chronic diseases such as T2DM, has not been fully established. Therefore, this study investigated the relationship between sdLDL-C and the occurrence and severity of CHD in Han Chinese patients with T2DM.

## 2. Methods

### 2.1. Study Population

We enrolled 3684 Han Chinese patients (2152 males, 1532 females) from Peking University International Hospital and Peking University People's Hospital from January 2012 to March 2015. The follow-up procedures were performed by experienced nurses or doctors every 6 months via telephone or face-to-face interviews. Major cardiovascular events (MACEs) are divided into cardiovascular mortality, nonfatal myocardial infarction (MI), nonfatal stroke, heart failure, hospitalized unstable angina, and noncoronary heart disease patients diagnosed with coronary heart disease. The longest follow-up time is 5 years.

All patients diagnosed with T2DM were selected based on the criteria set by the American Diabetes Association (ADA): (1) self-reporting to the clinician that he/she has a history of type 2 diabetes, (2) under current treatment of oral hypoglycemic medicine or insulin, (3) repeated fasting plasma glucose (FPG) greater than 7.0 mmol/L, or (4) glycated hemoglobin A1c (HbA1c) ≥ 6.5%.

The diagnostic criteria for patients with CHD were based on the coronary angiography (CAG) performed in our institution and defined as at least one major coronary artery occlusion or stenosis of more than 50%, and the severity of CHD was evaluated by the Gensini score (GS). According to the CAG results, diabetic patients were divided into CHD group and non-CHD group. Diabetes patients with CHD were divided into three groups according to their GS: low GS (GS ≤ 25), intermediate GS (GS: 26-40), and high GS (GS ≥ 41).

Exclusion criteria are as follows: (1) percutaneous coronary intervention within the previous three months, (2) acute coronary syndrome within the previous six months, (3) history of coronary artery bypass operation, (4) chronic heart failure, cardiomyopathy, or valvar heart disease, (5) pulmonary heart disease, (6) known inflammatory or infectious disease or confirmed or suspected cancer, or (7) severe liver or kidney dysfunction.

The present study complied with the Declaration of Helsinki and was approved by the Hospital Research Ethics Committee. Informed written consents were obtained from all patients enrolled in this study.

### 2.2. Conventional Clinical and Laboratory Indicator Tests

Blood samples were collected in the morning after at least 12 hours of fasting. All measurements were performed within 6 hours. FBG, homocysteine (HCY), hypersensitive C-reactive protein (hs-CRP), and serum lipid profiles, including TG, total cholesterol (TC), LDL-C, and HDL-C, were analyzed with a Beckman AU5832 analyzer (Beckman Coulter Inc., USA). Apolipoproteins A-1 (apoA1) and B (apoB) were measured by immunoturbidimetry (Daiichi Pure Chemicals Co., Ltd., Tokyo). Lipoprotein fraction Lp(a) in the serum samples was measured using latex-enhanced immunoturbidimetry Lp(a) kit (Roche Inc., Germany). Direct quantitative analysis of sdLDL-C assay was done using sdLDL-C reagent kits (Denka Seiken Co., Ltd. Japan). The hemoglobin A1c (HbA1c) was determined with high-performance liquid chromatography (Trinity Biotech Inc., USA).

### 2.3. Statistical Analyses

The distributions of all quantitative variables were analyzed using the one-sample Kolmogorov-Smirnov test. Normally distributed data were reported as the means ± standard deviations, and the differences between various groups were compared using the analysis of variance. Nonnormally distributed continuous data were reported as medians (interquartile ranges), and the differences between various groups were compared using the Kruskal-Wallis test. Categorical data was presented as percentage (%) and compared by the chi-squared test. The Mantel-Haenszel test for linear trend was used to detect whether the sdLDL-C levels were positively correlated with the CHD. The association of sdLDL-C with the presence and severity of CHD was analyzed using multivariate logistic regression adjusted for age, gender, body mass index (BMI), glucose, HbA1c, ApoB, ApoA1, TC, TG, HDL-C, LDL-C, Lp(a), hs-CRP, and HCY. The Kaplan-Meier method was used to estimate the event-free survival rates among groups. All data analyses were performed using SPSS software (version 22.0 for Windows, IBM Corp., USA). *p* < 0.05 was considered as statistically significant.

## 3. Results

### 3.1. Serum sdLDL-C Levels Are Increased in T2DM with CHD

A total of 3684 participants were enrolled in this study, including 2220 CHD patients and 1464 non-CHD patients.  Table 1 shows clinical characteristics and risk factors of all participants, including age, gender, glucose, HbA1c, blood-lipid indicators, Lp(a), hs-CRP, and HCY. The levels of ApoB, LDL-C, hs-CRP, HCY, and sdLDL-C in the CHD group were higher than those in the non-CHD group. The CHD group had a significantly lower apoA1 and HDL-C levels compared with the non-CHD group. There were no significant differences in other variables between the two groups (*p* > 0.05). We found that the serum sdLDL-C levels in the CHD group were significantly elevated compared with those in the non-CHD patients (0.80 (0.49) mmol/L vs. 0.70 (0.30) mmol/L, *p* < 0.001), indicating that the high level of serum sdLDL-C correlated with the presence of CHD. The results showed that high levels of sdLDL-C increased the risk of CHD in T2DM patients.

### 3.2. Serum sdLDL-C Levels Correlate with the Severity of CHD in T2DM Patients

According to the CAG results of each patient, the diabetic patients with CHD were further divided into multivessel disease group and high GS group to further evaluate the relationship between sdLDL-C and the severity of CHD (Table 2 and Table 3).

The patients with diabetes and CHD were then classified into single-vessel (*n* = 576), two-vessel (*n* = 656), and multiple-vessel disease (*n* = 988) groups (Table 2). We found a significant increase in the sdLDL-C levels in the multiple-vessel disease group [0.72 (0.24) mmol/L vs. 0.80 (0.24) mmol/L vs. 0.85 (0.52) mmol/L, *p* = 0.003]. The patients were also divided into three groups based on the GS tercile: low GS (≤25, *n* = 724), intermediate GS (26-40, *n* = 768), and high GS (≥41, *n* = 728) group (Table 3). The results indicated that the serum sdLDL-C levels in the high GS group were significantly higher than those in the other two groups [0.71 (0.29) mmol/L vs. 0.80 (0.27) mmol/L vs. 0.91 (0.53) mmol/L, *p* < 0.001].

After dividing diabetic patients with CHD into four groups according to the quartiles of sdLDL-C levels, the results showed a linear correlation between the level of sdLDL-C and the multiple-vessel disease group and high GS group. With the increase of serum sdLDL-C, the degree of coronary artery obstruction was more serious (Table 4) (*p* < 0.001).

To investigate the role of sdLDL-C in CHD, we also conducted a univariate and multivariate logistic regression analysis. All participants were divided into four quartiles of sdLDL-C levels, and the presence and severity of CHD in individuals with different sdLDL-C levels were assessed. In the univariate logistic regression analysis, sdLDL-C levels were associated with the presence and severity of CHD (CHD group vs. non-CHD group: OR = 2.515, 95% CI: 2.014-5.378, *p* = 0.019; multiple-vessel disease group vs. single-vessel disease group: OR = 3.781, 95% CI: 2.468–6.061, *p* < 0.001; high GS group vs. low GS group: OR = 2.891, 95% CI: 2.181–5.257, *p* < 0.001) (Table 5). The multivariate logistic regression was applied to adjust for age, gender, BMI, glucose, HbA1c, ApoB, ApoA1, TC, TG, HDL-C, LDL-C, Lp(a), hs-CRP, and HCY; the level of sdLDL-C remained to be independently associated with the presence and severity of CHD (CHD group vs. non-CHD group: OR = 2.257, 95% CI: 1.792–5.064, *p* = 0.023; multiple-vessel disease group vs. single-vessel disease group: OR = 3.288, 95% CI: 1.866–7.285, *p* = 0.026; high GS group vs. low GS group: OR = 2.554, 95% CI: 2.044–5.399, *p* = 0.022). These data together indicated that the level of sdLDL-C is positively correlated with the severity of CHD in T2DM patients.

### 3.3. sdLDL-C Levels and Cardiovascular Outcomes

**Figure 1:**
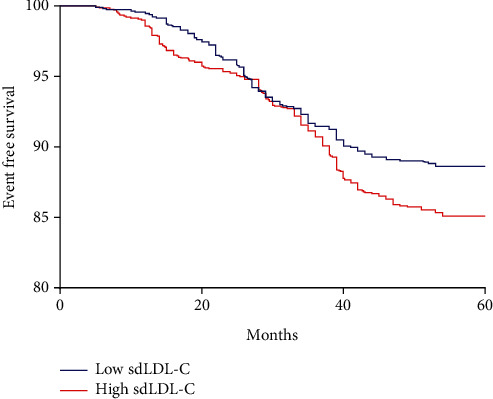
Kaplan-Meier curves according to median value of sdLDL-C.

During the follow-up period, there were 257 patients with data loss that did not enter the final follow-up analysis, and 484 (14.1%) of 3427 patients had MACEs (Table 6). All 3427 patients were divided into high sdLDL-C group and low sdLDL-C group according to their median sdLDL-C, and then, Kaplan-Meier analysis was performed on the high sdLDL-C and low sdLDL-C groups. The results showed that the incidence of MACEs in the high sdLDL-C group was higher than that in the low sdLDL-C group (Figure 1) (*p* = 0.002). We further divided the CHD group and the non-CHD group into low sdLDL-C and high sdLDL-C groups according to the median sdLDL-C to study the relationship between sdLDL-C and risk of MACEs. As shown in Table 6, the total incidence of MACEs in the high sdLDL-C group was significantly higher than that in the low sdLDL-C group in both the non-CHD and CHD groups, and the incidence of nonfatal MI in the CHD group was significantly higher compared with that in the low sdLDL-C group. These data indicate that sdLDL-C levels were associated with MACEs in T2DM patients.

## 4. Discussion

sdLDL-C, a subclass of LDL-C, is known to play a unique metabolic role in atherosclerosis [15–18]. In this study, we found the sdLDL-C levels were significantly associated with CHD occurrence and severity in T2DM patients. These findings suggest that serum sdLDL-C may be a potential biomarker in predicting the risk of MACEs [19, 20].

Previous studies have confirmed that high concentrations of sdLDL-C may be a risk factor for CHD [21–24]. It was reported that sdLDL-C is related to increased carotid intima media thickness and carotid plaque progression in different populations [25]. In an 11-year follow-up study of 11,419 volunteers, 1158 participants who developed CHD showed an average sdLDL-C concentration of 43.5 mg/dL [26]. In addition, the sdLDL-C levels were positively associated with diabetes, arterial hypertension, and increased BMI. In a Multi-Ethnic Study of Atherosclerosis (MESA), high levels of sdLDL-C were associated with the development of CHD. The top sdLDL-C quartile showed higher risk of incident CHD (hazard ratio, 2.41; *p* = 0.0037) compared with those in the bottom quartile, but not associated with T2DM [27]. However, Hsu et al. reported that the average sdLDL-C values in their subjects with impaired glucose tolerance (43.7 mg/dL) and those with diabetes mellitus (47.5 mg/dL) were higher than the average sdLDL-C value of the subjects with normal blood sugar and demonstrated positive associations between the sdLDL-C level and atherosclerotic and subclinical diabetes status in middle-aged Taiwanese without a history of CVD or diabetes mellitus [28]. The results of Hsu et al. support our findings on sdLDL-C and CHD. In order to better study the role of sdLDL-C levels in T2DM patients with CHD, we used a large Chinese cohort for this study. We found that the level of sdLDL-C in T2DM patients was related to the presence of CHD confirmed by the angiography. In addition, to further study the relationship between sdLDL-C and CHD severity in diabetic patients, we used GS system to conduct multiple subgroup analysis and our study shows that the higher the GS score in diabetic patients, the higher the serum sdLDL-C level.

Besides, the present study has several limitations. First, only two centers were involved in the research, which might have led to selective biases in the data results, and some of the conclusions should be verified in larger multicenter studies. Second, some data were missing and the serum sdLDL-C levels were not determined during the follow-up. However, our data still provide evidence that the incidence and severity of CHD increase with the increase of serum sdLDL-C level in patients with T2DM. In addition to this, there is an increased risk of cardiovascular events in the future.

## 5. Conclusion

In conclusion, our study indicated that the increased sdLDL-C is an independent predictor of CHD and is related to the severity of CHD, and it is a certain ability to predict cardiovascular events. These findings suggest that sdLDL-C is a crucial biomarker for the prediction of the occurrence and severity of CHD and cardiovascular events in T2DM patients.

## Figures and Tables

**Table 1:** Baseline characteristics in type 2 diabetic patients.

Variables	Total	CHD group	Non-CHD group	*p* value
*N* (%)	3684	2220 (60.26%)	1464 (39.74%)	—
Age (years)	58.61 ± 9.32	59.48 ± 9.62	56 89 ± 8.98	0.296
Male (%)	2152 (58.41%)	1324 (59.64%)	828 (56.56%)	0.422
BMI (kg/m^2^)	26.16 ± 3.6	26.51 ± 3.2	25.81 ± 3.3	0.203
Glucose (mmol/L)	7.56 ± 2.10	7.85 ± 2.12	7.37 ± 2.08	0.082
HbA1c (%)	7.42 ± 1.33	7.61 ± 1.42	7.35 ± 1.35	0.089
ApoB (mg/dL)	82.78 ± 28.70	88.15 ± 29.87	74.65 ± 27.39	<0.001
ApoA1 (mg/dL)	140.24 ± 32.67	116.83 ± 29.67	175.83 ± 35.04	<0.001
Total cholesterol (mmol/L)	4.44 (1.33)	4.46 (1.72)	4.41 (0.98)	0.737
Triglycerides (mmol/L)	1.32 (0.88)	1.32 (0.82)	1.32 (0.94)	0.987
HDL-C (mmol/L)	1.02 (0.46)	0.86 (0.29)	1.31 (0.39)	<0.001
LDL-C (mmol/L)	2.71 (0.95)	2.87 (1.35)	2.61 (0.61)	<0.001
Lp(a) (nmol/L)	39.25 (39.75)	39.71 (48.37)	38.09 (33.46)	0.576
hs-CRP (mg/L)	2.10 (2.31)	3.10 (2.90)	1.63 (1.56)	0.028
HCY (mumol/L)	11.06 (7.74)	11.99 (10.22)	9.55 (5.53)	<0.001
sdLDL-C (mmol/L)	0.74 (0.37)	0.80 (0.49)	0.70 (0.30)	<0.001

Data are reported as the means ± SD or *n* (%), median (interquartile ranges). SD: standard deviation; BMI: body mass index; HbA1c: hemoglobin A1c; apoB: apolipoprotein B; apoA1: apolipoprotein A1; HDL-C: high-density lipoprotein cholesterol; LDL-C: low-density lipoprotein cholesterol; Lp(a): lipoprotein (a); hs-CRP: hypersensitive C-reactive protein; HCY: homocysteine; sdLDL-C: small dense low-density lipoprotein cholesterol. Statistical analysis was performed with the ANOVA or Kruskal-Wallis test and with chi-squared test for categorical variables.

**Table 2:** Baseline characteristics in type 2 diabetic patients with multiple-vessel disease of coronary heart disease patients.

Variables	1 vessel	2 vessels	≥3 vessels	*p* value
*N* (%)	576 (25.95%)	656 (29.55%)	988 (44.50%)	—
Age (years)	58.67 ± 10.72	59.79 ± 11.21	59.28 ± 9.88	0.700
Male (%)	336 (58.3%)	384 (58.5%)	604 (61.1%)	0.661
BMI (kg/m^2^)	26.23 ± 2.9	26.57 ± 3.2	27.54 ± 3.5	0.156
Glucose (mmol/L)	8.07 ± 2.32	7.61 ± 2.09	7.51 ± 2.11	0.095
HbA1c (%)	7.77 ± 1.47	7.53 ± 1.39	7.59 ± 1.41	0.212
ApoB (mg/dL)	67.65 ± 21.74	85.22 ± 20.72	101.77 ± 29.43	<0.001
ApoA1 (mg/dL)	111.87 ± 31.49	115.85 ± 30.24	118.87 ± 26.41	0.071
Total cholesterol (mmol/L)	4.35 (1.62)	4.51 (1.70)	4.68 (1.85)	0.037
Triglycerides (mmol/L)	1.32 (0.82)	1.38 (0.83)	1.30 (0.78)	0.723
HDL-C (mmol/L)	0.89 (0.32)	0.85 (0.30)	0.82 (0.27)	0.537
LDL-C (mmol/L)	2.68 (1.05)	2.83 (1.00)	2.99 (1.34)	0.014
Lp(a) (nmol/L)	37.80 (40.61)	41.51 (49.94)	39.47 (45.54)	0.189
hs-CRP (mg/L)	5.60 (8.03)	4.41 (7.12)	3.10 (6.57)	0.002
HCY (mumol/L)	11.60 (8.14)	12.69 (10.37)	12.00 (11.53)	0.078
sdLDL-C (mmol/L)	0.72 (0.24)	0.80 (0.24)	0.85 (0.52)	0.003

Data are reported as the means ± SD or *n* (%), median (interquartile ranges). SD: standard deviation; BMI: body mass index; HbA1c: hemoglobin A1c; apoB: apolipoprotein B; apoA1: apolipoprotein A1; HDL-C: high-density lipoprotein cholesterol; LDL-C: low-density lipoprotein cholesterol; Lp(a): lipoprotein (a); hs-CRP: hypersensitive C-reactive protein; HCY: homocysteine; sdLDL-C: small dense low-density lipoprotein cholesterol. Statistical analysis was performed with the ANOVA or Kruskal-Wallis test and with chi-squared test for categorical variables.

**Table 3:** Baseline characteristics in type 2 diabetic patients with Gensini score of coronary heart disease patients.

Variables	Low GS	Intermediate GS	High GS	*p* value
*N* (%)	724 (32.62%)	768 (34.59%)	728 (32.79%)	—
Age (years)	59.65 ± 10.02	60.11 ± 10.43	58.72 ± 8.91	0.776
Male (%)	420 (58.01%)	464 (60.42%)	440 (60.44%)	0.744
BMI (kg/m^2^)	26.46 ± 3.28	26.26 ± 3.19	26.89 ± 3.47	0.540
Glucose (mmol/L)	8.11 ± 2.31	7.72 ± 2.02	7.64 ± 2.10	0.181
HbA1c (%)	7.81 ± 1.48	7.55 ± 1.42	7.57 ± 1.39	0.197
ApoB (mg/dL)	82.17 ± 26.06	86.67 ± 22.58	90.78 ± 30.37	0.006
ApoA1 (mg/dL)	115.25 ± 29.75	113.02 ± 31.57	117.03 ± 26.89	0.412
Total cholesterol (mmol/L)	4.37 (1.40)	4.49 (1.41)	4.73 (1.83)	0.012
Triglycerides (mmol/L)	1.32 (0.76)	1.25 (0.66)	1.37 (0.83)	0.332
HDL-C (mmol/L)	0.88 (0.33)	0.86 (0.30)	0.84 (0.24)	0.698
LDL-C (mmol/L)	2.77 (1.04)	2.89 (1.07)	3.09 (1.45)	0.026
Lp(a) (nmol/L)	38.54 (40.71)	40.09 (45.17)	41.17 (51.39)	0.664
hs-CRP (mg/L)	3.40 (7.08)	4.55 (11.32)	4.06 (8.39)	0.476
HCY (mumol/L)	11.35 (10.15)	12.27 (9.29)	12.69 (11.79)	0.045
sdLDL-C (mmol/L)	0.71 (0.29)	0.80 (0.27)	0.91 (0.53)	<0.001

Data are reported as the means ± SD or *n* (%), median (interquartile ranges). SD: standard deviation; BMI: body mass index; HbA1c: hemoglobin A1c; apoB: apolipoprotein B; apoA1: apolipoprotein A1; HDL-C: high-density lipoprotein cholesterol; LDL-C: low-density lipoprotein cholesterol; Lp(a): lipoprotein (a); hs-CRP: hypersensitive C-reactive protein; HCY: homocysteine; sdLDL-C: small dense low-density lipoprotein cholesterol. Statistical analysis was performed with the ANOVA or Kruskal-Wallis test and with chi-squared test for categorical variables.

**Table 4:** Linear relationship between small dense low-density lipoprotein cholesterol levels and severity of coronary heart disease.

sdLDL-C (mmol/L)	1 vessel	2 vessels	≥3 vessels	*p*	Low GS	Intermediate GS	High GS	*p*
0.12–0.58	215	160	173	<0.001	267	161	124	<0.001
0.58–0.80	147	203	212		180	256	160	
0.81–1.07	103	151	296		129	189	210	
1.07–2.31	111	142	307		146	162	234	

sdLDL-C: small dense low-density lipoprotein cholesterol; GS: Gensini score. Statistical analysis was performed with the Mantel-Haenszel test for linear trend.

**Table 5:** Odd ratios of CHD, multiple-vessel disease, and high GS in relation to quartiles of small dense low-density lipoprotein.

Variables	sdLDL-C (mmol/L)			
	<0.58	0.58-0.74	0.75-0.95	>0.95
*CHD*				
Model 1^a^				
Odds ratio (95% CI)	1.00 (ref.)	1.868 (1.062-5.064)	3.168 (1.560-7.616)	2.515 (2.014-5.378)
*p* value	—	0.225	0.129	0.019
Model 2^b^				
Odds ratio (95% CI)	1.00 (ref.)	1.696 (1.226-5.626)	1.587 (1.198-6.876)	2.205 (1.617-5.145)
*p* value	—	0.398	0.198	0.042
Model 3				
Odds ratio (95% CI)	1.00 (ref.)	1.626 (1.162-5.464)	1.742 (1.201-6.564)	2.257 (1.792-5.064)
*p* value	—	0.341	0.143	0.023
*Multiple-vessel disease*				
Model 1^a^				
Odds ratio (95% CI)	1.00 (ref.)	1.852 (1.298–4.012)	3.411 (2.127-8.813)	3.781 (2.468–6.061)
*p* value	—	0.156	0.078	<0.001
Model 2^b^				
Odds ratio (95% CI)	1.00 (ref.)	2.112 (1.353–4.003)	3.177 (1.708-9.242)	3.461 (1.786-6.586)
*p* value	—	0.107	0.129	0.014
Model 3^c^				
Odds ratio (95% CI)	1.00 (ref.)	2.002 (1.310–4.212)	3.158 (1.801–6.745)	3.288 (1.866-7.285)
*p* value	—	0.121	0.009	0.026
*High GS*				
Model 1^a^				
Odds ratio (95% CI)	1.00 (ref.)	1.607 (1.145-5.020)	2.965 (2.050–5.237)	2.891 (2.181–5.257)
*p* value	—	0.101	0.045	<0.001
Model 2^b^				
Odds ratio (95% CI)	1.00 (ref.)	1.872 (1.210-6.512)	2.458 (1.602-8.031)	2.622 (1.991-5.521)
*p* value	—	0.199	0.171	0.028
Model 3^c^				
Odds ratio (95% CI)	1.00 (ref.)	1.602 (1.010-5.112)	2.402 (1.619-7.712)	2.554 (2.044-5.399)
*p* value	—	0.171	0.121	0.022

sdLDL-C: small dense low-density lipoprotein cholesterol; GS: Gensini score; CHD: coronary heart disease; CI: confidence interval. ^a^Univariate model. ^b^Adjusted for age, sex, and body mass index. ^c^Additionally adjusted for hemoglobin A1c, apolipoprotein B, apolipoprotein A1, high-density lipoprotein cholesterol, low-density lipoprotein cholesterol, lipoprotein (a), hypersensitive C-reactive protein, and homocysteine.

**Table 6:** sdLDL-C levels are related to the incidence of MACEs.

MACEs	Non-CHD group		*p*	CHD group		*p*
	Low sdLDL-C (*n* = 687)	High sdLDL-C (*n* = 679)		Low sdLDL-C (*n* = 1024)	High sdLDL-C (*n* = 1037)	
CHD	46 (6.70%)	48 (7.07%)	0.431	—	—	
Cardiovascular deaths	0 (0%)	2 (0.29%)	0.247	21 (2.05%)	26 (2.51%)	0.293
Nonfatal MI	5 (0.73%)	10 (1.47%)	0.144	61 (5.96%)	87 (8.39%)	0.020
Nonfatal strokes	2 (0.29%)	7 (1.03%)	0.086	32 (3.13%)	39 (3.76%)	0.251
Heart failure	2 (0.29%)	3 (0.44%)	0.494	9 (0.88%)	14 (1.35%)	0.210
Hospitalized unstable angina	9 (1.31%)	18 (2.65%)	0.056	21 (2.05%)	22 (2.12%)	0.517
Total	64 (9.32%)	88 (12.96%)	0.020	144 (14.06%)	188 (18.13%)	0.007

sdLDL-C: small dense low-density lipoprotein cholesterol; CHD: coronary heart disease; MACEs: major cardiovascular events. Statistical analysis was performed with the chi-squared test for categorical variables.

## Data Availability

The datasets used and/or analyzed during the current study are available from the corresponding author on reasonable request.
